# Spot Detection for Laser Sensors Based on Annular Convolution Filtering

**DOI:** 10.3390/s23083891

**Published:** 2023-04-11

**Authors:** Lingjiang Li, Maolin Li, Weijun Sun, Zhenni Li, Zuyuan Yang

**Affiliations:** 1 Guangdong Key Laboratory of IoT Information Technology, School of Automation, Guangdong University of Technology, Guangzhou 510006, China; lagcyp@msn.com (L.L.); lml617658@163.com (M.L.); gdutswj@gdut.edu.cn (W.S.); lizhenni2012@gmail.com (Z.L.); 2 Key Laboratory of Intelligent Detection and the Internet of Things in Manufacturing (GDUT), Ministry of Education, Guangzhou 510006, China; 3 Guangdong-Hong Kong-Macao Joint Laboratory for Smart Discrete Manufacturing, Guangzhou 510006, China

**Keywords:** laser sensor, spot detection, image processing, ACF

## Abstract

Spot detection has attracted continuous attention for laser sensors with applications in communication, measurement, etc. The existing methods often directly perform binarization processing on the original spot image. They suffer from the interference of the background light. To reduce this kind of interference, we propose a novel method called annular convolution filtering (ACF). In our method, the region of interest (ROI) in the spot image is first searched by using the statistical properties of pixels. Then, the annular convolution strip is constructed based on the energy attenuation property of the laser and the convolution operation is performed in the ROI of the spot image. Finally, a feature similarity index is designed to estimate the parameters of the laser spot. Experiments on three datasets with different kinds of background light show the advantages of our ACF method, with comparison to the theoretical method based on international standard, the practical method used in the market products, and the recent benchmark methods AAMED and ALS.

## 1. Introduction

With the development of laser sensor technology, lasers are widely used in modern society [1], such as in medicine, measurement, communication, and industry, due to their characteristics of directional luminescence, high brightness, pure color, high energy density, high monochromaticity, and high coherence [2,3]. For example, in the medical field [4,5], laser coagulation is currently mainly used in the repair of retinal detachment. Laser has the advantages of high precision, good hemostatic effect, and minimal invasiveness. In the industry field [6], laser is increasingly occupying an important position. Laser-based satellite communications are necessary, which can provide 10 to 100 times higher data transfer rates than radios, due to the higher bandwidth [7].

The quality of laser directly affects the laser sensors. The study of laser beam quality has always been an active field of laser science [8,9]. At the same time, the detection of laser beam quality is inseparable from the laser spot, so the detection and analysis of the laser spot has become an important research topic [10,11]. In laser applications, precise measurement of the spot parameters is necessary. Therefore, it is of great significance to find a suitable method to detect and analyze the parameters of the laser spot. At present, there are mainly two kinds of methods for detecting the parameters, including the mechanical scanning method and the optical imaging method [12,13].

Regarding the mechanical scanning method, it is popular, traditional, and suitable for applications with little requirement for detection time. The method in [14] proposes a diffraction measurement technique for the spot beam using the moving slit method. The method proposed in [15] uses the knife-edge method to measure the resolution of aerial images. However, since this kind of method utilizes the mechanical scanning scheme, it is time-consuming to obtain the parameters of the laser, and it is hard to give the variance of the laser in a timely fashion. In contrast to the traditional mechanical scanning method, the optical imaging method has faster measurement speed and higher instant measurement accuracy. Due to the usage of the fast area array CCD for the detector, it can improve the quality of laser products as a whole. Therefore, the latter attracts special attention for measuring the spot size of laser beams. In fact, it can simultaneously and quickly measure the light intensity distribution in the two-dimensional direction of the beam cross-section, which is suitable for measuring the characteristic parameters of the laser beam shape [16]. At present, this scheme is used in many methods for detecting laser spot parameters. For example, for speckle denoising and edge detection, the method proposed in [17] combines median filter and Gaussian filter to eliminate speckle noise to improve the method of detecting the center of the speckle. The proposed method of edge detection in [18] is based on the least squares error method and subpixel edge-detection algorithm. The method proposed in [19] performs edge detection with adjacent dispersion, which avoids threshold selection, anisotropy in convolution computation, and edge discontinuity. The method proposed in [20] is an unbiased non-local mean fuzzy C-means method based on local Zernike moments for edge detection on spot images. The method in [21] proposes to use surrounding pixel values as local context to estimate the probability of a pixel belonging to an edge, which considers using surrounding pixels for edge detection. The method proposed in [22] utilizes a contour-based segmentation scheme by employing the Canny algorithm, which combines various operations such as morphology and reconstruction to remove noise before performing edge detection.

The method in [23] proposes to perform image segmentation based on edge ratio statistics and adaptive threshold to perform edge detection. The method proposed in [24] is an edge-detection algorithm based on multi-scale, which can detect the weak edges of images. The method proposed in [25] uses a small-area weak-edge signal-detection method. This method detects edges by projecting boundary signals in the column direction to increase the gradient of edges. The method proposed in [26] is an improved Canny algorithm based on morphology, where the used morphological filtering not only removes image noise but also strengthens the protection of image edges, and the double-detection thresholds are used for further segmentation to obtain final edge points. The method proposed in [27] provides a fractional-order adaptive p-Laplace equation image edge-detection algorithm. This method preserves the texture and details of the image while removing noise to detect image edges. The method proposed in [28] designs a quantum image edge-detection scheme with enhanced quantum representation of digital images and multi-directional grayscale morphology for the detection of image edges.

### Related Work and Our Contribution

For spot center detection, the method in [29] proposes a reaction–diffusion (RD) system as the main computational framework to robustly find laser spot centers. Lately, the least squares method or the circle-fitting algorithm have been widely discussed in regards to performing contour fitting and then estimating the center coordinates of the spot [30,31,32,33]. In recently work, the method proposed in [34] develops the Gaussian-weighted adaptive threshold scheme to detect the position coordinates of the spot center. The method proposed in [35] uses a random sampling scheme to select the candidate center of a circle and then updates it iteratively. An iterative two-zone contraction method based on baseline strategy is proposed in [36].

For spot-shape fitting, a method combining Gram Schmidt orthogonalization and least square elliptic fitting is proposed in [37], where only two interferograms are needed for each wavelength. The method proposed in [38] develops the supervised learning model based on domain randomization technique. The method proposed in [39] designs a fractional Fourier transform (FRFT)-based scheme, which uses the continuity of FRFT to extract the edge of the image to detect the spot shape. The method in [40] adds a concave and convex arc determination algorithm on the basis of Hough transform to improve the accuracy of spot-shape fitting. The method proposed in [41] provides a projection-invariant pruning algorithm to fit the light spot shape. Methods in [42,43,44,45,46] use edge curvature information and gradient information to segment the curve into arcs, and use the convexity and concavity of the arc to fit the shape of the spot.

Specifically, in this paper, we propose a spot detection method for laser sensors based on annular convolution strips. Our method comprehensively utilizes the statistical information of the gray value of the spot and the Gaussian distribution characteristics of the spot to improve the measurement accuracy and robustness of the spot parameters. It is mainly based on the statistical properties of the spot image, and the statistical moment formula is employed to calculate the ellipse center and the tilt angle, and they are updated iteratively, together with the corresponding ROI region. Then, according to the Gaussian distribution characteristics of the laser spot, the annular convolution strip is generated for the convolution operation. Finally, a feature similarity index is designed to estimate the parameters of the elliptical spot, including the long axis and the short axis.

## 2. Materials and Methods: Spot Detection Using Annular Convolution Filtering

### 2.1. The Gaussian Laser Spot

In our method, we first generate the background-free laser spot image using Matlab software. For calculating the focused laser beam, we use the Rayleigh Formula (1) to calculate the Rayleigh length, which is determined by the beam waist radius $\omega_{0}$ and wavelength λ.


(1) $$z_{0}=\frac{\pi \omega_{0}^{2}}{\lambda }$$

At present, the most commonly used laser wave lengths in the market are 1064 nm, 532 nm, and 355 nm. In our method, we focus on $\omega_{0}$ = 1 mm and λ = 532 nm. Based on the Rayleigh length $z_{0}$, one can further calculate the beam radius W(z) of the light spot during the propagation process by

(2) $$W(z)=\omega_{0}\sqrt{1+{\left(\frac{z}{z_{0}}\right)}^{2}}$$

where z represents the position distance of the propagation process.

Then, the points in the Gaussian beam image can be represented by

(3) $$I(z,r)=I_{0}*\exp(-2*\frac{r^{2}}{W{\left(z\right)}^{2}})$$

where r denotes the physical distance of any point and the central point, and $I_{0}$ represents the maximum light intensity (we set $I_{0}=220$ in the experiments). Denote η to be the ratio of the total distance and the total pixel number, which is a known parameter of the sensor. Then, in the case that the coordinate of the central point is (0,0), one can rewrite (3) for any point with pixel position coordinate (x,y) by


(4) $$I(z,x,y)=I_{0}*\exp(-2\eta^{2}*\frac{x^{2}+y^{2}}{W{\left(z\right)}^{2}})$$

For simplicity, we focus on z=2 in this paper, and the Gaussian beam image with a general central point $\left(\tilde{x},\tilde{y}\right)$ is finally given by


(5) $$I(x,y)=I_{0}*\exp(-2\eta^{2}*\frac{{\left(x-\tilde{x}\right)}^{2}+{\left(y-\tilde{y}\right)}^{2}}{W{\left(2\right)}^{2}})$$

The formula in (5) is used to generate the spot for experiments in Section 3.

### 2.2. ROI Determination

In order to measure the laser spot parameters more accurately, one often needs to first determine the ROI. We utilized the difference method to remove the background information inside and outside the spot, and obtained an image with only light spot information finally. Similar to the method in [47], the implementation steps of finding ROI are as follows.

(1) Search the initial central coordinate O: list the gray value of each pixel of the given image I, let the variable G be the maximum gray value and set the corresponding pixel position as the initial central coordinate $O(\tilde{x},\tilde{y})$;

(2) Determine the initial ROI area: find the flag pixels whose gray values closely equal to G×13.5% in I, where the attenuating ratio 13.5% is empirical and also used in [47]. For each pixel in this flag set, count the pixel number between it and O, and denote the maximum and minimum of the number set by $\tilde{m}$ and $\tilde{n}$, respectively. Finally, set $m=2\tilde{m}$ and $n=2\tilde{n}$ to be the length and width of the initial ROI area;

(3) Update the central coordinate $O(\tilde{x},\tilde{y})$: according to the grayscale information in the initial ROI, we used the following Formula (6) to calculate, approximately, the center based on the statistical characteristics of the signal:

(6) $$\left\{ \begin{array}{l} \hat{x}=\frac{\sum_{x=\tilde{x}-\tilde{m}}^{\tilde{x}+\tilde{m}}\sum_{y=\tilde{y}-\tilde{n}}^{\tilde{y}+\tilde{n}}xI(x,y)}{\sum_{x=\tilde{x}-\tilde{m}}^{\tilde{x}+\tilde{m}}\sum_{y=\tilde{y}-\tilde{n}}^{\tilde{y}+\tilde{n}}I(x,y)}\\ \hat{y}=\frac{\sum_{x=\tilde{x}-\tilde{m}}^{\tilde{x}+\tilde{m}}\sum_{y=\tilde{y}-\tilde{n}}^{\tilde{y}+\tilde{n}}yI(x,y)}{\sum_{x=\tilde{x}-\tilde{m}}^{\tilde{x}+\tilde{m}}\sum_{y=\tilde{y}-\tilde{n}}^{\tilde{y}+\tilde{n}}I(x,y)} \end{array}\right.$$

where I(x,y) is the gray value of the image at coordinate (x,y). It is notable that the order of the pixels in I starts from left to right, up to down, i.e., the pixel at the left and up position corresponds to the coordinate (1,1). The updated central coordinate is $\tilde{x}=p(\hat{x}),\;\tilde{y}=p(\hat{y})$, where p(x) denotes the closest integer to x. Reset $G=I(\tilde{x},\tilde{y})$;

(4) Determine the final ROI: repeat steps (2) and (3) until convergence.

### 2.3. Annular Convolution Strip

In the process of convolution operation on the general image, one often strips the convolution kernel along the coordinate axes. However, for the laser spot image, it suffers from overlap of the edge information and the background light, resulting in low accuracy and poor robustness of the parameters for detecting the spot. In this paper, according to the energy distribution characteristics of the laser spot, we propose an annular convolution strip to detect the contour of the laser spot.

Firstly, according to the statistical characteristics of the signal, the gray information of the pixels in the ROI is used to calculate the tilt angle θ of the spot, and the corresponding formula is as follows [47].

(7) $$\theta =\arctan\frac{A+B}{C}$$

where $\left\{ \begin{array}{l} A=I(x,y)(\sum_{x=\tilde{x}-\tilde{m}}^{\tilde{x}+\tilde{m}}{\left(x-\tilde{x}\right)}^{2}-\sum_{y=\tilde{y}-\tilde{n}}^{\tilde{y}+\tilde{n}}{\left(y-\tilde{y}\right)}^{2})\\ B=\sqrt{A^{2}+4{\left(\sum_{x=\tilde{x}-\tilde{m}}^{\tilde{x}+\tilde{m}}\sum_{y=\tilde{y}-\tilde{n}}^{\tilde{y}+\tilde{n}}I(x,y)(x-\tilde{x})(y-\tilde{y})\right)}^{2}}\\ C=2\sum_{x=\tilde{x}-\tilde{m}}^{\tilde{x}+\tilde{m}}\sum_{y=\tilde{y}-\tilde{n}}^{\tilde{y}+\tilde{n}}I(x,y){\left(x-\tilde{x}\right)}^{2}{\left(y-\tilde{y}\right)}^{2} \end{array}\right.$, and $\left(\tilde{x},\tilde{y}\right)$ denotes the central coordinate.

**Figure 1:**
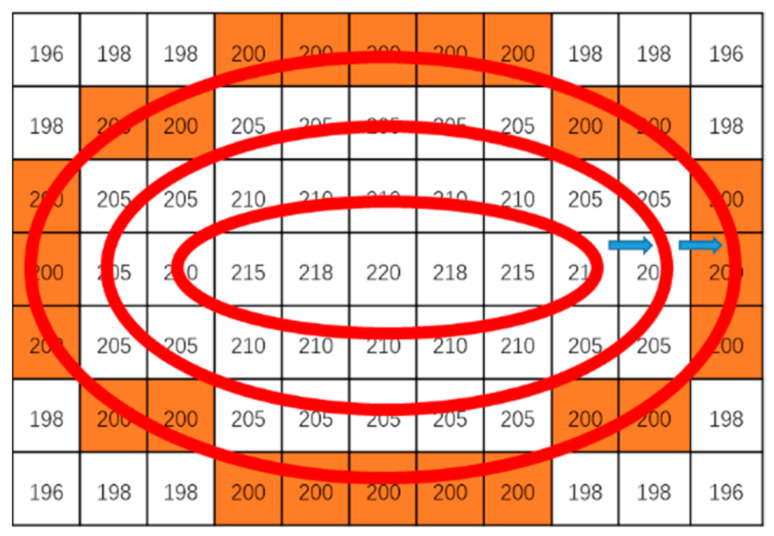
Annular convolution strip.

Then, we construct the following annular convolution strip according to parameters about the center $\left(\tilde{x},\tilde{y}\right)$ and the angle θ:

(8) $$f(x,y,a,b)=\frac{{\left((x-\tilde{x})\cos\theta +(y-\tilde{y})\sin\theta \right)}^{2}}{a^{2}}+\frac{{\left((y-\tilde{y})\cos\theta +(x-\tilde{x})\sin\theta \right)}^{2}}{b^{2}}$$

where (x,y) denotes the coordinate of the pixel point, a denotes the long axis, b denotes the short axis (b≤a), and θ denotes the tilt angle. In the model (8), when f(x,y,a,b)=1, the annular convolution strip is generated for convolution operation. Figure 1 shows the convolution operation that is carried out in the ROI of the spot image. Since the pixels of the spot image are discrete, one needs to utilize an error parameter ε to determine the exact pixel coordinates for convolution operation (see the model (9)).


(9) $$\phi (a_{ik},b_{ik})=\left(\underset{\left\{x,y\right\}}{\arg}{\left(f\left(x,y,a_{ik},b_{ik}\right)-1\right)}^{2}\leq \varepsilon \right)$$

Take Figure 1, for example, to show the elements of ϕ in (0). Denote the center of I with the largest number 220 by $O(\tilde{x},\tilde{y})$ and the smallest red strip by $f_{1}$. Then, by using (9) under a very small parameter ε, the annular strip coordinates set $\phi_{1}$ corresponding to $f_{1}$ obtains 12 elements, i.e., $\phi_{1}^{i}={\tilde{\phi }}_{1}^{i}+(\tilde{x},\tilde{y}),\forall i$, where ${\tilde{\phi }}_{1}$ is below:


$${\tilde{\phi }}_{1}=\{(-3,0),(-2,1),(-2,-1),(-1,1),(-1,-1),(0,1),(0,-1),(1,1),(1,-1),(2,1),(2,-1),(3,0)\}$$

**Figure 2:**
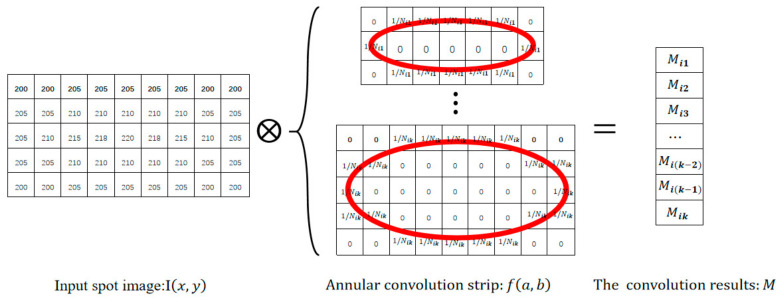
The process of the convolution operation.

Once ϕ is obtained, a projection function g is used to generate the convolution factor $\hat{\phi }$, where any element of $\hat{\phi }=g(\phi )$ is often a constant. Regarding the process of the convolution operation, it is given in the model (10) and further shown in Figure 2. Actually, we first determine the location of the generated annular convolution strip through the center O, then determine the direction of the strip according to the tilt angle θ, and finally perform the convolution operation in the ROI area $\hat{I}$ of the spot image. As the gray values of $\hat{I}$ are the same to the corresponding part of I, we do not discriminate between them in some circumstances for the uniform usage of the pixel coordinates.


(10) $$M_{ik}=\hat{I}⊗\hat{\phi }(a_{ik},b_{ik})$$

It is notable that the convolution operation is helpful to estimate the attenuation attribute of the laser. Regarding the attenuation attribute, one often needs only a small number of feature points to measure it. Based on the positions of these points in the guided spot, we select the needed feature points ${\hat{M}}_{ik}$ from the convolution points $M_{ik}$, where the projection function g is set to be $1/N_{ik}$ and $N_{ik}=\left|\phi (a_{ik},b_{ik})\right|$ is the number of pixels in the annular convolution strip. Finally, ${\hat{M}}_{ik}$ can be calculated by:


(11) $${\hat{M}}_{ik}=\frac{\sum_{\left(x,y)\in \phi (a_{ik},b_{ik}\right)}I(x,y)}{N_{ik}}$$

We further design a feature similarity index to determine the contour of the input spot. This index reflects the Euclidean distance between the selected convolution result ${\hat{M}}_{i}=\left[{\hat{M}}_{i1},\cdots ,{\hat{M}}_{ik}\right]$ and the standard and known spot feature $\tilde{M}=\left[{\tilde{M}}_{1},\cdots ,{\tilde{M}}_{k}\right]$, and it is calculated by the following Equation (12).


(12) $$S_{i}=\sqrt{({\hat{M}}_{i}-\tilde{M})({\hat{M}}_{i}-\tilde{M}{)}^{\prime }}$$

Then, the estimated contour of the input spot is determined according to the minimum value of S. The proposed algorithm is summarized in Algorithm 1.
**Algorithm 1:** The proposed ACF algorithm.Input: original laser spot image with background light;Output: The long axis $\hat{a}$ and the short axis $\hat{b}$ of the estimated spot;Step 1: Calculate the ROI, the central coordinate, and tilt angle by using the method in Section 3.2;Step 2: Obtain the optimal ratio of the short axis and the long axis using the following iteration, where a=l, b=1 in the initialization;   Set i=1;   While b<a      Calculate $r_{i}=b/a$;      Set k=1, $\bar{b}=b$;   While k<K   Let $a_{ik}=a,b_{ik}=b$, calculate $M_{ik}$ by Equation (11);   Update a=a+Δa;       Update $b=ar_{i}$;       Update k=k+1;   End while   Calculate the feature similarity $S_{i}$ by Equation (12);   Update a=l;   Update $b=\bar{b}+1$;      Update i=i+1;   End while   Output the optimal ratio $r_{i}$ corresponding to the minimum value of *S*;Step 3: Fitting a new ellipse using the results in Step 1 and Step 2, where the ratio of the energy in this ellipse and that in ROI is chosen as the widely used value 86.5% (see [48]);Step 4: Output the long axis $\hat{a}$ and the short axis $\hat{b}$ of the ellipse in Step 3.

**Remarks** **1.**
 l  *is often set by the approximate pixel number between the center and the point with 80% of the maximum gray value in the direction of the tilt angle,*  K  *denotes the maximum strip number, and*  Δa  *is a positive integer, such as 5 without loss of generality.*

In this section, the Gaussian feature of the laser is introduced first, and then the ROI of the whole image is determined. Finally, the proposed algorithm ACF is derived for detecting the laser spot in ROI. In next section, numerical experiments are given to show the performance of our ACF, including the used data introduction, the parameter sensitivity analysis, and the results with comparison to the related methods

## 3. Results

In this section, we conduct experiments on three datasets and perform a comparative analysis with four related methods. In our experiments, each compared method is implemented using MATLAB R2011b installed in a personal computer with Intel(R) Celeron(R) 2.4-GHz CPU, 2-GB memory, and the Microsoft Windows 10 operational system.

### 3.1. Datasets

#### 3.1.1. Standard Dataset

**Figure 3:**
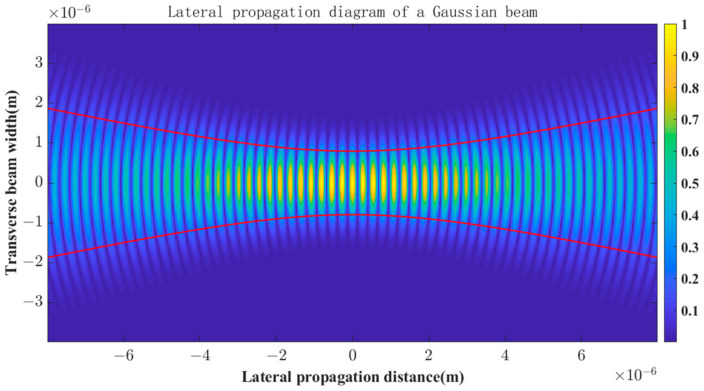
Lateral propagation diagram of the Gaussian beam.

**Figure 4:**
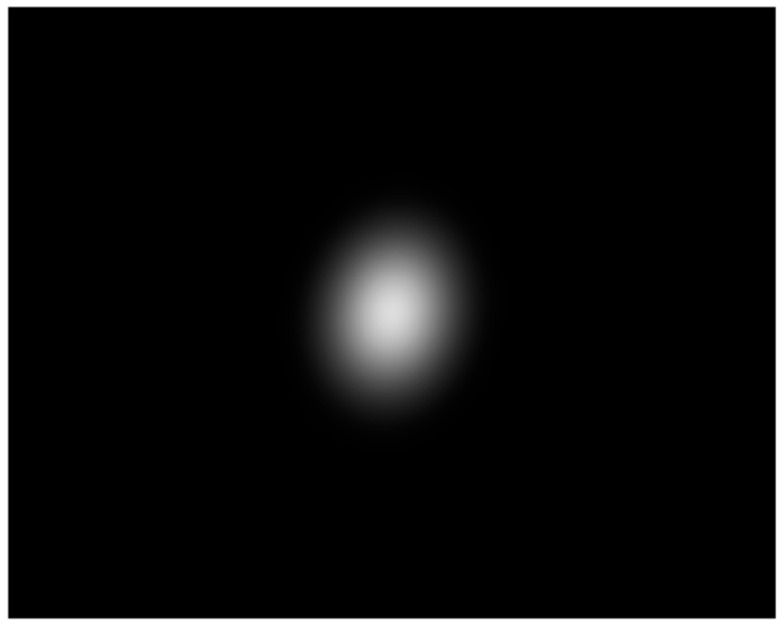
Spot image without background light.

Based on the steps in Section 3.1, we generated a Gaussian spot image using the Matlab software. Figure 3 is a diagram of the lateral propagation of a fundamental mode Gaussian beam. Figure 4 is the generated simulation spot image, where $I_{0}=220$, and $\tilde{x}=640,\tilde{y}=512$. The elements of the real spot feature vector $\tilde{M}$ in Equation (12) are 206.03, 200.82, 194.93, 189.17, 183.19, 176.68, 168.77, 161.12, 153.18, 145.26, ⋯.

#### 3.1.2. Test Dataset

To generate the test dataset, the standard Gaussian spot in (1) was mixed with different background light images. The following three datasets were tested in our experiments. Dataset 1 was obtained by superimposing the standard spot data and the uniform light background. Dataset 2 was obtained by superimposing the standard spot data with the fluorescent light background. Dataset 3 was obtained by superimposing the standard spot data with the natural light background.

### 3.2. Compared Methods

To verify the performance of our proposed method, we compared it with the following four methods.

Theoretical method (TM) [48]: This method is mainly used for processing the spot without background light. It uses the second moment of light intensity to define the beam width. It first uses a two-dimensional area array detector to measure the light intensity distribution of the laser beam on a certain transverse mode plane. Then, the first-order intensity moment and second-order intensity moment of the laser beam are obtained according to the light intensity distribution, and, finally, the long and short axes of the beam are calculated.

Practical method (PM): This method is widely used in market products with the typical software Anbeam. It uses the second-order moment of light intensity and the definition of power flux to define the beam width. It first performs adaptive background filtering on the collected light spots. Then, the center coordinates of the light spot are calculated by the second moment method of light intensity. Finally, the least squares method is used to fit the ellipse of the spot, and then calculate the long and short axes of the beam.

AAMED Method [44]: This method proposes an ellipse-detection algorithm based on arc adjacency matrix. Firstly, the extracted edge lines are divided into elliptic arcs, and then a direct arc adjacency matrix AAM is constructed. Secondly, through bidirectional traversal of AAM, all possible combinations of arcs that could be real candidates for ellipses are obtained, together with the cumulative matrix CM based on the accumulation factor CF. Finally, a synthetic formula is given to calculate the verification score to estimate the long and short axes of the beam.

ALS Method [45]: This method uses LSD algorithm to connect and group arc-support line segments and generate the initial ellipse candidate set. Then, it clusters the ellipses that may come from the same ellipse candidate set, and eliminate the ellipse with low quality. At last, the long and short axes of the estimated ellipse are obtained.

### 3.3. Parametric Sensitive Analysis

In this subsection, we analyze the performance of the proposed method under different parameters. Since the used strip number and select parameters *ε* of circular convolutions may affect the values of the long and short axes of the spot, they are mainly tested, and the performance index includes $E_{a}=\hat{a}-a_{0}$ and $E_{b}=\hat{b}-b_{0}$, where $\hat{a}$, $\hat{b}$ are the long axis and short axis, respectively, estimated by using our ACF, and $a_{0},b_{0}$ are the real long axis and short axis, respectively.

**Figure 5:**
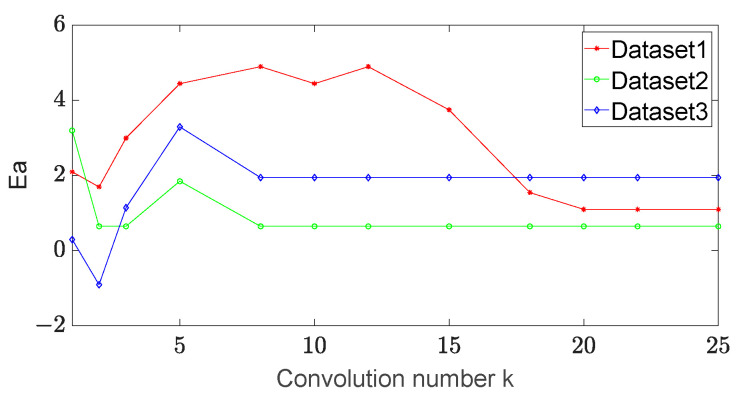
The error of the long axis vs. the convolution number in different datasets.

**Figure 6:**
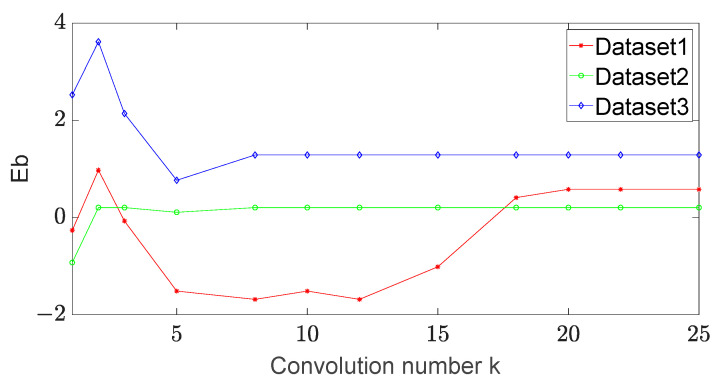
The error of the short axis vs. the convolution number in different datasets.

(1) The number of convolution strip varies in the set {5, 10, 15, 20, 25}. The entire convolution operation is performed for each update step, and the annular convolution operation is performed on the three test datasets above. When the number of convolution strips on the spot image is changed, the errors of the long and short axes are detected. The experimental results are shown in Figure 5 and Figure 6.

From these figures, we find that the errors of the long axis and the short axis of dataset 1 change greatly with the increase in the number of convolutions at the beginning. When the number of convolutions becomes 20, the errors of both the long and short axes of dataset 1 tend to be stable. When the number of convolutions becomes 10, the errors of the long and short axes of dataset 2 and dataset 3 tend to be stable. We set the number of convolutions to 20 in the experiments for comparison.

**Figure 7:**
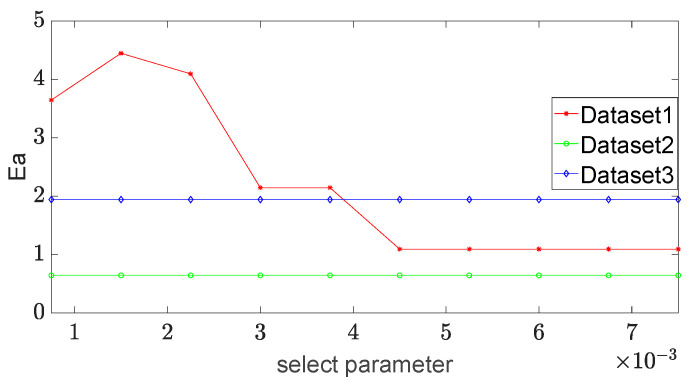
The error of the long axis vs. select parameter in different datasets.

**Figure 8:**
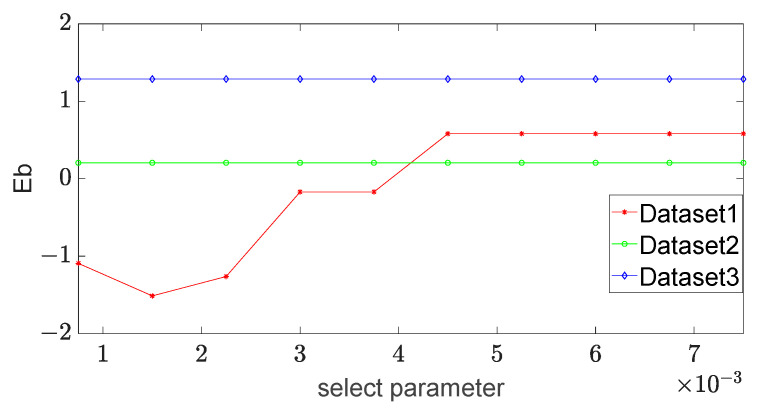
The error of the short axis vs. select parameter in different datasets.

(2) Before performing the convolution operation on the spot image, it is necessary to set the select parameter *ε* to determine the exact pixels chosen to be calculated. If the value of the selection parameter is changed, the number of the selected pixels in the spot image is changed, and the corresponding errors of the long and short axes could be changed. They are detected and the influence of this parameter on the stability of the long and short axes is analyzed. The select parameter varies in {0.00075, 0.0015, 0.00225, 0.003, 0.00375, 0.0045, 0.00525, 0.006, 0.00675, 0.0075}, and the experimental results are shown in Figure 7 and Figure 8.

It can be seen from Figure 7 and Figure 8 that the errors of the long axis and short axis in dataset 1 change greatly and they are robust in the other two datasets. When the select parameter is 0.0045, the errors of both the long and short axes of dataset 1 tend to be stable. We set this parameter to 0.0045 in the experiments for comparison.

### 3.4. Compared Results

In this subsection, we conduct experiments on three datasets and evaluate our algorithm with the laser spot feature (i.e., long axis and short axis), and the results are compared with that of the related methods. We first use the parameters that generate the simulated spot to obtain the standard data, which are used for comparison. To demonstrate the generality of our method, we use different background light in different dataset. The experimental results are show in Table 1, Table 2 and Table 3.

Table 1 shows the comparison of spot parameter measurement results of dataset 1 for five different methods, from which one can see that all the spot parameters measured by ACF have the smallest difference from the standard data. Regarding the long axis and short axis, the differences of ACF are 1.09 and 0.57, the differences of TM are 1.59 and 7.37, the differences of PM are 9.2 and 1.75, the differences of AAMED are 7.93 and 0.75, and the differences of ALS are 10.97 and 3.03, respectively. For the simulated spot images, the ACF method can fit the elliptical spot shape more accurately in the environment with background light. The main reason is that the method ACF uses the annual convolution filtering to fit the shape of the spot, which effectively protects the grayscale information of the spot and improves the measurement accuracy of the spot parameters.

Regarding our method, after $\hat{M}$ is calculated by the Equation (11), the elements of its optimal feature are 205.31, 200.75, 196.57, 190.05, 182.47, 175.10, 167.38, 160.32, 152.69, 144.87, ⋯, where the feature similarity value 4.87 is the smallest in the feature similarity vector S calculated by Equation (12). Clearly, the elements of this optimal feature are close to that of the standard feature $\tilde{M}$. Thus, our method has good performance.

Table 2 shows the comparison of spot parameter measurement results of dataset 2 for five different methods, from which one can see that on the long axis and short axis, the differences between ACF and the standard data are 0.64 and 0.2, the differences of TM are 3.05 and 8.71, the differences of PM are 10.1 and 2.05, and the differences of ALS are 10.53 and 3.1, respectively. As for AAMED, it does not work in this dataset, as it cannot find the needed ellipse due to the interference of the background light.

For our method, the elements of the optimal feature selected from $\hat{M}$, which is calculated by the Equation (11), are 205.89, 200.50, 195.34, 189.26, 182.17, 175.71, 168.29, 160.71, 152.94, 145.05, ⋯, where the feature similarity value 2.72 is the smallest in the vector S. These values are close to that of the standard feature $\tilde{M}$, implying the competitive performance of our method.

Table 3 shows the comparison of spot parameter measurement results of dataset 3 for five different methods, from which one can see that on the long axis and short axis, the differences between ACF and the standard data are 1.94 and 1.28, the differences of TM are 1.3 and 6.83, the differences for PM are 13.4 and 5.25, and the differences of ALS are 11.64 and 4.54, respectively. Again, due to the interference of the background light, AAMED does not work in this dataset. The experimental results demonstrate that ACF has the best accuracy in measuring the spot parameters.

As for the details of our method, the elements of the optimal feature selected from $\hat{M}$ are 205.38, 200.23, 195.33, 190.07, 182.95, 175.68, 168.27, 161.14, 153.44, 144.56, ⋯, where the feature similarity value 2.31 is the smallest in the vector S. Again, one can see that these values are close to that of the standard feature $\tilde{M}$, clearly reflecting the effectiveness of our ACF.

## 4. Discussion and Conclusions

In this paper, we propose a novel spot detection for laser sensors based on annular convolution filtering (ACF). We comprehensively use the gray value information of the light spot and the Gaussian distribution characteristics of the light spot to improve the measurement accuracy and robustness of the light spot parameters. We first use the grayscale information of the light spot to calculate the central coordinate and tilt angle of the light spot, and further determine the ROI. Then, based on the Gaussian distribution characteristics of the laser spot, the feature of the spot is obtained by using annual convolution filtering, and the similarity index is used to determine the parameters of spot. Finally, a new ellipse is constructed as the estimated spot. We conducted experiments on three datasets with different background light. At the same time, we compare it with four related methods. Experimental results show the effectiveness of our method. Specifically, the parameters of the long axis and the short axis of the annular spot estimated by our method are much closer to the real parameters than that of the compared methods, due to the development of statistical features of the image.

It is worth noting that the ellipse estimated by PM used in the current commercial system is often much bigger than the real one due to the interference of background light. The proposed method has advantages in detecting the laser beam characteristics under the interference. These characteristics play critical parts in generating a robust beam profiler, which can monitor the working status of the laser products.

Generally, the study of the spot detection method is very important, especially in the case that there is background light. Actually, an excellent beam profiler is necessary for the laser user to process the material precisely, as it can reduce the irreversible damage to the material by analyzing the quality of the laser in prior. It leads to the necessity of our study, which can detect more precisely the unknown spot under the background light.

In this study, we focus the detection method based on the image processing. Our method performs the best during the compared methods. In future, it could be extended further to process more complex background light, such as the dynamic light, the strong light, and so on.

## Figures and Tables

**Table 1:** Standard data and the results of the compared methods on dataset 1.

	Standard Data	ACF	TM	PM	AAMED	ASL
Long axis a (nm)	158	159.09	156.41	167.2	165.93	168.97
Short axis b (nm)	132	132.57	124.63	133.75	132.75	135.03

**Table 2:** Standard data and the results of the compared methods on dataset 2.

	Standard Data	ACF	TM	PM	AAMED	ASL
Long axis a (nm)	158	158.64	154.95	168.1	/	168.53
Short axis b (nm)	132	132.2	123.29	134.05	/	135.1

**Table 3:** Standard data and the results of the compared methods on dataset 3.

	Standard Data	ACF	TM	PM	AAMED	ASL
Long axis a (nm)	158	159.94	156.7	171.4	/	169.64
Short axis b (nm)	132	133.28	125.17	137.25	/	136.54

## Data Availability

No new data were created.
